# The microbial communities and metabolic profiles of follicular fluid in patients with premature ovarian insufficiency

**DOI:** 10.3389/fendo.2024.1447397

**Published:** 2025-01-07

**Authors:** Wei Wang, Mingming Shu, Jianhua Li, Qihang Wang, Wendan Zhang, Ye Wang, Yiming Guo, Yanbin Cheng, Honghong Jiang, Chunlan Song, Yuan Liu, Wei Shang

**Affiliations:** ^1^ Department of Obstetrics and Gynecology, The Seventh Medical Center of Chinese People's Liberation Army (PLA) General Hospital, Beijing, China; ^2^ Department of Obstetrics and Gynecology, Chinese PLA General Hospital, Beijing, China; ^3^ Department of Obstetrics and Gynecology, The Sixth Medical Center of Chinese PLA General Hospital, Beijing, China; ^4^ Faculty of Pediatrics, The Seventh Medical Center of Chinese PLA General Hospital, Beijing, China; ^5^ Department of Biology, Kenneth P. Dietrich School of Art and Science, University of Pittsburgh, Pittsburgh, PA, United States

**Keywords:** premature ovarian insufficiency, 16S rDNA amplicon sequencing, metabolomics, ABC transporters, citrate cycle (TCA cycle)

## Abstract

**Introduction:**

Premature ovarian insufficiency (POI) is a condition characterized by ovarian dysfunction occurring before the age of 40, and its etiology is multifactorial, including genetic, immunological, infectious, environmental, and iatrogenic factors, with over half of the cases remaining unexplained. Whether the microbial communities and metabolites in follicular fluid, which is the direct microenvironment for oocyte survival, are related to POI has not been reported.

**Methods:**

In this study, Follicular fluid samples of 26 patients with POI and 27 controls with a normal ovarian reserve were collected and analyzed using 16S rDNA sequencing and untargeted metabolomics. Conjoint analysis was performed to identify key microbial communities and metabolites that might be involved in POI.

**Results:**

Patients with POI exhibited significant alterations in microbial richness and diversity and metabolic profile in their follicular fluid. The downregulation of ABC transporters and upregulation of the citrate cycle (TCA cycle) might be critical for the development and progression of POI. G-Rhodopseudomonas and g-Caulobacter were identified as key microbial genera, while L-aspartic acid, citrate, isoleucine, and cytidine were identified as key metabolites.

**Discussion:**

These findings offer novel insights into the pathogenesis of POI and might pave the way for improved clinical outcomes for individuals with POI.

## 1 Introduction

Premature ovarian insufficiency (POI) is a clinical syndrome characterized by a decrease in ovarian activity before the age of 40 years, with menstrual disorders such as amenorrhea or irregular menstruation and high levels of gonadotropins and low estrogen. The root cause of POI at any stage may be a reduction in the number of primordial follicles, cell apoptosis or an increase in follicular destruction and an ineffective response to gonadotropin stimulation (1, 2). Genetic factors, immunity, medical factors, inflammation, etc., are known risk factors for POI. However, more than 50% of POI cases have unknown causes (3–6).

Recently, it was found that the intestinal microbiota can affect ovarian function (7). The intestinal microbiota in POI patients changed significantly, as indicated by a decrease in the richness of Firmicutes, Bacteroides and Faecalibacterium and an increase in the richness of Proteobacteria and Bacteroides. An ecological imbalance and a reduction in the diversity of the intestinal microbiota could reduce the activity of β-glucuronidase and lead to a decrease in estrogen activity (8). There was a correlation between the intestinal microbiota and serum estrogen, follicle-stimulating hormone, luteinizing hormone, and anti-Müllerian hormone levels (9–11). Intestinal microbiota imbalance is also associated with autoimmune POI. An imbalance in the intestinal microbiota not only affects the activation of B lymphocytes and the production of autoantibodies but also induces the abnormal activation of innate immune cells, leading to the upregulation of proinflammatory cytokines. Disruption of the balance between the intestinal microbiota and the immune system can exacerbate the progression of POI (12).

Follicular fluid (FF), which serves as the direct microenvironment for oocyte development, is not sterile. It is composed of plasma exudates and local secretions from the ovary and is rich in monosaccharides, proteins, steroid hormones, cytokines, and growth factors. Therefore, FF is considered an excellent growth medium for microorganisms (13). Studies have shown that the composition and richness of the microbiota in follicular fluid significantly vary among infertile women, and these changes are also influenced by age (13–15). Dysregulation of lipid metabolism in human follicular fluid is closely related to ovarian reserve function, and changes in arachidonic acid metabolism might be related to oocyte development, leading to a decline in fertility among individuals with diminished ovarian reserve (DOR) (16). The metabolomics of follicular fluid confirmed metabolic disorders in DOR individuals. The differentially abundant metabolites were mainly enriched in the choline pathway, with significant differences in the concentrations of pregnenolone-3-glucuronide sulfate and 2-hydroxyl-4-androstene-3-one sulfate (17).

The composition and richness of the follicular fluid microbiota in patients with POI have not been extensively studied. The underlying mechanisms responsible for these changes remain unknown. This study sought to compare the differences in follicular fluid microbiota and metabolites between patients with POI and their control group (CG) using 16S rDNA amplicon sequencing and untargeted metabolic technology. By correlating differential microbial communities in follicular fluid with differentially abundant metabolites, we aimed to identify key microbial communities and metabolites that regulate ovarian microecology leading to POI. These findings will provide new insights into improving the clinical outcomes of POI patients.

## 2 Materials and methods

### 2.1 Participant recruitment

This study recruited 53 patients who underwent IVF-ET treatment at the Reproductive Medicine Center of the 7th Medical Center of Chinese PLA General Hospital between September 2022 and June 2023, including 26 patients in the POI group and 27 patients in the CG. According to the European Society of Human Reproduction and Embryology (ESHRE) guidelines, the inclusion criteria for patients with POI were as follows: < 40 years of age, menstrual irregularities (amenorrhea or oligomenorrhea > 4 months) with a basic FSH level > 25 IU/L for two consecutive times > 4 weeks apart (1). The inclusion criteria for the CG were as follows: < 35 years of age, normal ovarian reserve function, and normal chromosome karyotype. The exclusion criteria for both groups were the use of hormone drugs and antibiotics within the previous 3 months, alcohol intake, sexually transmitted infections and urinary tract infections, amenorrhea caused by organic disease of the uterus, and abnormal thyroid function, and a family history of genetic disorders. In addition, diseases that may influence the microbiome, such as inflammatory bowel disease, endometriosis, and ovarian endometriomas were also excluded. This study was approved by the Ethical Committee of the 7th Medical Center of Chinese PLA General Hospital (Ethics Number: 2022-58), and all participants enrolled in this study signed informed consent.

### 2.2 Sample collection

All patients received an appropriate ovarian hyperstimulation treatment protocol for the first time (18). According to the downregulation results, follicle-stimulating hormone (FSH)/human menopausal gonadotropin (HMG) was given to promote the development of multiple follicles, and B-mode ultrasound monitoring was performed to observe follicle development. When the average diameter of the dominant follicle was 14-18 mm and the E_2_ level of each follicle was 150-250 pg/ml, patients were injected with 5000-10000 IU Human Chorionic Gonadotropin (hCG) or 250 mg of recombinant hCG to induce ovulation, and oocytes and follicular fluid were retrieved 24-36 hours later. To minimize the risk of vaginal or cervical contamination during sample collection, it was important to avoid contact between the negative pressure needle and the cervix or vaginal wall. Only follicular fluid from the first dominant follicle free of blood cells was collected using a disposable sterile tube and immediately placed in liquid nitrogen for cryopreservation until testing.

### 2.3 Analysis of follicular fluid 16S rDNA amplicon sequencing

Database construction and sequencing: The genomic DNA of the follicular fluid was extracted using an Omega Mag-Bind Soil DNA Kit (M5635-00, Omega, USA) according to the manufacturer’s protocol. The concentration of the DNA was detected using a Nanodrop 2000 (Thermo Fisher, USA). The V3-V4 hypervariable regions of the diluted genome (1 ng/μl) were amplified using a gradient PCR instrument (T100, Bio-Rad, USA) with the specific primers 338F-806R and Barcode. The forward primer sequence was ACTCCTACGGGAGGCAGCA, while the reverse primer sequence was GGACTACHVGGGTWTCTAAT. Library construction was performed with the TruSeq^®^ DNA PCR-Free Sample Preparation Kit (Illumina, USA). The constructed library was sequenced on an Illumina NovaSeq 6000 sequencing system (Illumina, USA) with a PE250 read length.

Sequence Analysis: To study the microbial composition diversity, we employed QIIME2 to perform amplicon sequence variant (ASV) clustering and annotation with the Silva 138 database (https://www.arb-silva.de/). Alpha diversity analysis was conducted using the Wilcoxon rank-sum test. The species composition of the two groups was compared using principal coordinate analysis (PCoA). ANOSIM was employed to assess whether there were significant differences in microbial community structure between groups, thereby determining the meaningfulness of the grouping. Wilcoxon rank sum tests were utilized to analyze beta diversity based on Bray–Curtis distances. Additionally, STAMP and linear discriminant analysis (LDA) effect size (LEfSe) were used to identify differential microbial communities. PICRUSt software was used to perform Kyoto Encyclopedia of Genes and Genomes (KEGG) functional prediction. Furthermore, based on the prediction results, the LEfSe algorithm was utilized to analyze intergroup differential functions.

### 2.4 Untargeted metabolomics profiling

Metabolite Extraction: After the samples were slowly thawed at 4°C, an appropriate amount of each sample was added to precooled methanol/acetonitrile/aqueous solution (2:2:1, v/v), vortexed, ultrasonicated at low temperature for 30 min, incubated at -20°C for 10 min, and centrifuged at 14000 g at 4°C for 20 min, after which the supernatant was vacuum dried. During mass spectrometry, 100 μL of acetonitrile solution (acetonitrile:water =1:1, v/v) was added to redissolve the dried samples. The mixture was again vortexed and centrifuged at 14,000 g at 4°C for 15 minutes, and the resulting supernatant was then used for further analysis.

### 2.5 Liquid chromatography–mass spectrometry analysis

After being separated by Vanquish LC ultrahigh-performance liquid chromatography (UHPLC), the samples were subjected to mass spectrometry using a Q Exactive series mass spectrometer (Thermo Fisher, USA) and detected by electrospray ionization (ESI) in both positive and negative ion modes (19, 20). Throughout the analysis process, the samples were placed in an automatic sample injector at 4°C. To avoid fluctuations in instrument detection signals, the samples were analyzed continuously in random order. QC samples were inserted into the sample queue to monitor and evaluate the stability of the system and the reliability of the experimental data.

### 2.6 Data analysis

The original data were converted into the mzXML format by ProteoWizard, and then XCMS software was used for peak alignment, retention time correction and peak area extraction. The extracted data were first subjected to metabolite structure identification and data preprocessing, followed by quality control assessment, which included total ion chromatography (TIC) of the QC samples, principal component analysis (PCA) of the total samples, Pearson correlation analysis of the QC samples, Hotelling’s T2 test, multivariate control chart (MCC) and relative standard deviation (RSD) of the QC samples. Finally, data analysis was performed, including the identification level, quantity, and composition of the follicular fluid metabolites, multidimensional statistical analysis based on orthogonal partial least squares discriminant analysis (OPLS-DA), differentially abundant metabolite selection (OPLS-DA Variable importance in the projection (VIP) > 1 and P value < 0.05 as the screening criteria), differentially abundant metabolite hierarchical clustering analysis, KEGG pathway annotation and analysis, etc.

### 2.7 Conjoint analysis of 16S rDNA amplicon sequencing and metabolites in follicular fluid

First, the correlation coefficients between differential microbial communities (LEfSe LDA > 2 and p value < 0.05) and metabolites (VIP > 1 and t test p value < 0.05) selected were analyzed using the Spearman statistical method. Subsequently, a correlation network analysis was conducted on the differential microbial communities and metabolites with absolute correlation coefficients between [0.5, 1] and p values < 0.05, aiming to identify the key nodes in the network, and then Cytoscape 3.5.1 software was used to plot the correlation network diagram. A correlation hierarchical clustering analysis of differential microbial communities and metabolites was performed, and the results were displayed as a heatmap using the R 3.4.2 Heatmap package. Finally, based on the coanalysis of differential microbial communities and metabolites, key microbial communities, metabolites, and metabolic regulatory pathways of POI were identified to explore the interactions between microbial communities and metabolites from multiple perspectives.

## 3 Results

### 3.1 The demographic and clinical characteristics of patients with POI and CG

A total of 53 participants were recruited, including 26 patients in the POI group and 27 patients in the CG. The comparison of baseline demographic data is presented in **Table 1**. No significant disparities were observed between the two groups regarding the duration of infertility, type of infertility, body mass index (BMI), or basal estradiol (bE2) level. Conversely, POI patients were notably older than those in the control group, exhibiting pronounced increases in both basal FSH and basal luteinizing hormone (LH) levels. Additionally, anti-Müllerian hormone (AMH) levels were substantially decreased, which was mirrored by a marked decrease in the number of retrieved oocytes, metaphase II (MII) oocytes, two pronuclear (2PN) fertilized oocytes, and day 3 high-quality embryos. These findings indicated a substantial impairment in ovarian reserve function among POI patients.

**Table 1:** The demographic and clinical characteristics of patients with POI and CG.

Characteristics	POI	CG	p-value
Number of patients	26	27	
Age (year)	36.24 ± 3.22***	31.96 ± 2.68	<0.001
Duration of infertility(year)	3.85 ± 2.03	3.41 ± 1.82	0.412
Type of infertility, n (%)			
Primary	16(61.5)	21(77.8)	0.198
Secondary	10(38.5)	6(22.2)	
BMI (kg/m2)	22.03 ± 2.37	23.57 ± 3.24	0.056
AMH (ng/mL)	0.56 ± 0.42***	2.70 ± 1.43	<0.001
bFSH (IU/L)	27.32 ± 2.19***	6.98 ± 3.86	<0.001
bLH (IU/L)	7.44 ± 2.92**	4.60 ± 3.71	0.003
bE2 (pg/mL)	68.77 ± 94.94	45.94 ± 48.15	0.272
P (ng/mL)	0.30 ± 0.14*	0.50 ± 0.37	0.016
Oocytes retrieved (n)	2.73 ± 1.85***	14.44 ± 6.87	<0.001
MII oocytes (n)	2.54 ± 1.73***	11.96 ± 5.07	<0.001
2PN Fertilizations (n)	2.15 ± 1.49***	9.41 ± 3.88	<0.001
Day3 High-quality embryos (n)	1.31 ± 1.29***	5.59 ± 2.85	<0.001

POI, Premature ovarian insufficiency; CG, control group; BMI, Body mass index; AMH, anti-Mullerian hormone; bFSH, basic follicle-stimulating hormone; bLH, basic luteinizing hormone; bE2, basic estrogen; P, progesterone; *p ≤ 0.05 **p ≤ 0.005 ***p ≤ 0.001..

### 3.2 Analysis of 16S rDNA amplicon sequencing

The composition of the follicular fluid microbiota in patients with POI significantly changed.

**Figure 1:**
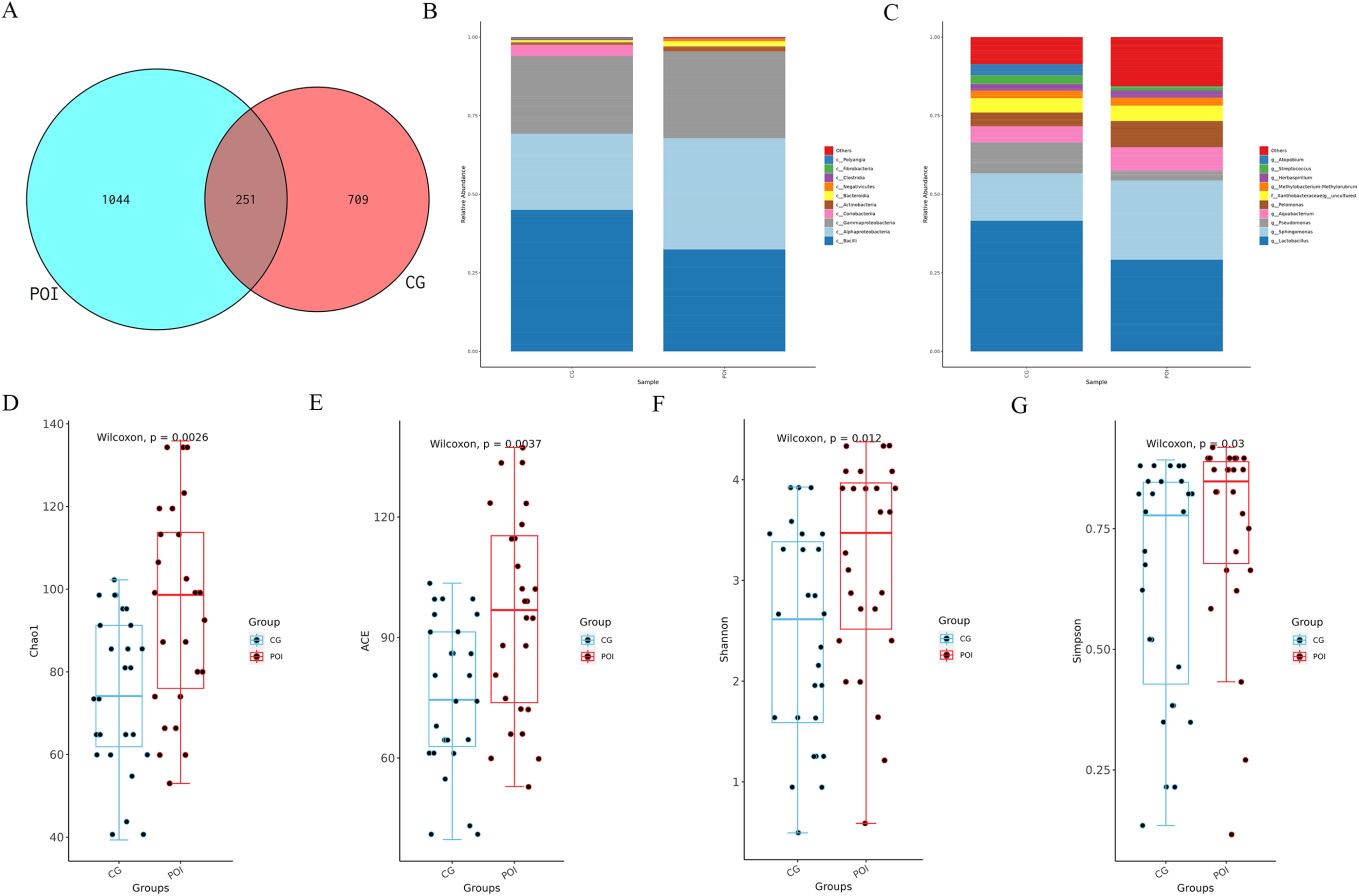
ASVs, species annotation and alpha diversity analysis of follicular fluid microbial communities in patients with POI and CG. **(A)** Venn diagram showed the number of shared and unique ASVs between POI (n =26) and CG (n =27) groups; **(B)** Top 10 major microbial annotations at the phylum level; **(C)** Top 10 major microbial annotations at the genus level; **(D-G)** showed that the alpha diversity indices (Chao1, ACE, Shannon, and Simpson), revealed significant statistical differences (P < 0.05); (CG, Control group; POI, Premature ovarian insufficiency).

Using 16S rDNA amplicon sequencing technology, the composition of the follicular fluid microbiota in the POI and CG groups was analyzed. ASV clustering analysis revealed 251 shared ASVs between the two groups, with 1044 unique ASVs in the POI group and 709 unique ASVs in the CG group (**Figure 1A**). The annotation of these follicular fluid microbiota (**Figures 1B, C**) revealed that the top 10 major constituents of the follicular fluid microbiota at the phylum level were *Proteobacteria, Firmicutes, Actinobacteria, Bacteroidota, Fibrobacterota, Myxococcota, Cyanobacteria, Synergistota, Acidobacteriota, Campilobacterota*, etc. At the genus level, the top 10 microbial communities included *Lactobacillus, Sphingomonas, Pseudomonas, Aquabacterium, Pelomonas, f_Xanthobacteraceae|g_uncultured, Methylobacterium-Methylorubrum, Herbaspirillum, Streptococcus, and Atopobium*. Next, the community richness and diversity within the two groups of FF microbiota (i.e., alpha diversity) were analyzed. The results revealed a significant difference in the richness and diversity of the intragroup microbiota between the two groups (P < 0.05) (**Figures 1D–G**).

**Figure 2:**
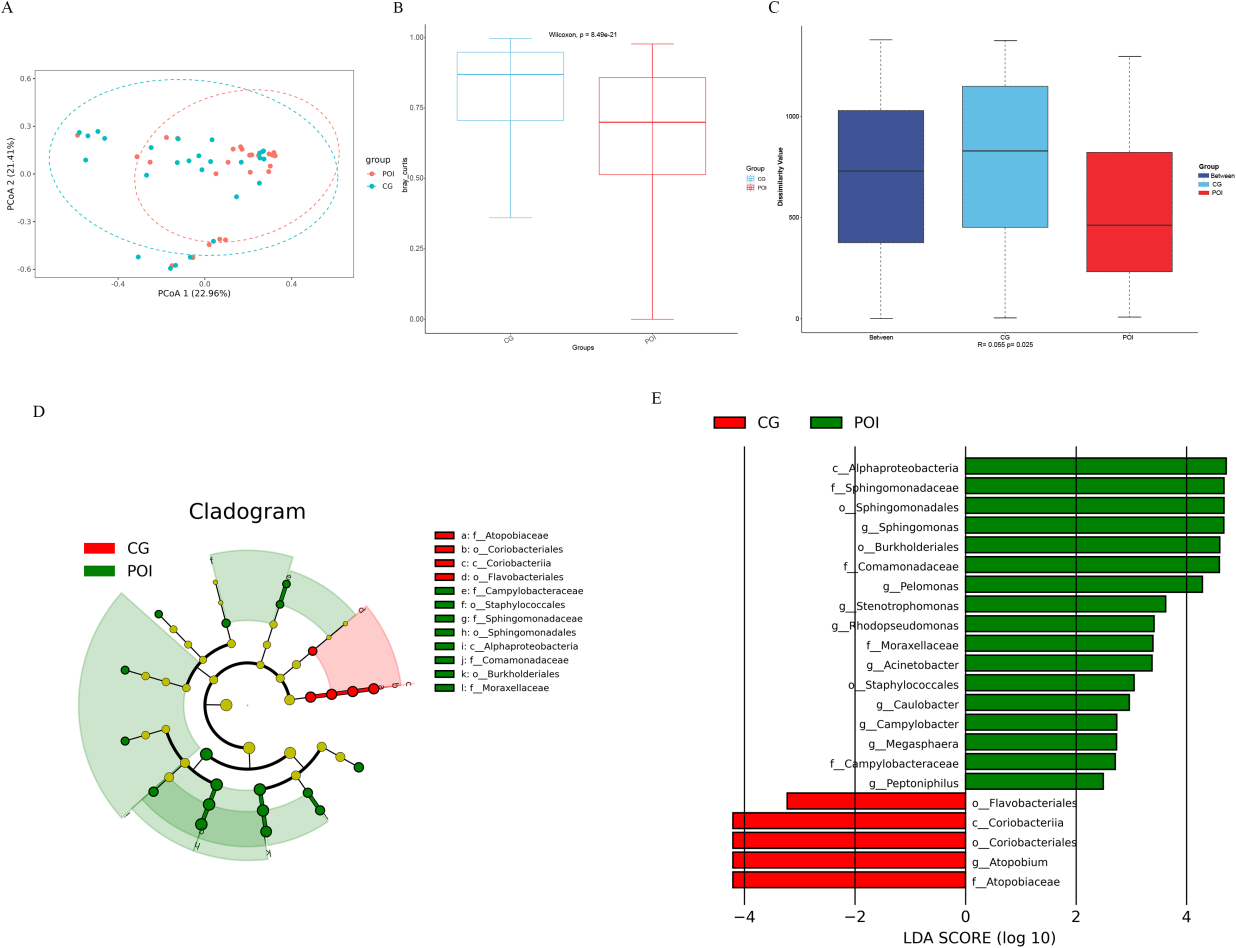
Beta diversity and LDA Effect Size (LEfSe) analysis of follicular fluid microbial communities in patients with POI and CG. **(A)** Bray Curtis-Principal Co-ordinates Analysis (PCoA), **(B)** Bray Curtis-Beta diversity analysis (Wilcoxon, P = 8.49e-21), **(C)** Bray Curtis-Anosim analysis (R = 0.055, P = 0.03); **(D)** Cladogram, the legend on the right showed the differential microbial communities at the phylum, class, order and family level; **(E)** LDA value distribution histogram showed the different levels of differential microbial communities from phylum to genus.

Beta diversity analysis based on the Bray–Curtis distance also revealed significant differences in the follicular fluid microbiota between the two groups (Wilcoxon, P = 8.49e-21). Moreover, the intergroup differences were significantly greater than the intragroup differences (Anosim R = 0.055, P = 0.03) (**Figures 2A–C**). LEfSe analysis (screening criteria: LDA value > 2, p value < 0.05) revealed 22 differential microbial communities at different levels between the two groups (**Supplementary Table S1**), as shown in the cladogram (**Figure 2D**) and the LDA value distribution histogram (**Figure 2E**). Among these, there were 10 differential microbial communities at the genus level. The richness of Sphingomonas, Pelomonas, Rhodopseudomonas, Stenotrophomonas, Acinetobacter, Caulobacter, Megasphaera, Campylobacter, and Peptoniphilus was significantly greater in the POI group than in the CG group. Additionally, the presence of Atopobium was significantly greater in the CG group. Among the top 10 major microbial communities, Pelomonas and Sphingomonas were enriched in the POI group, while Atopobium was enriched in the CG group.

**Figure 3:**
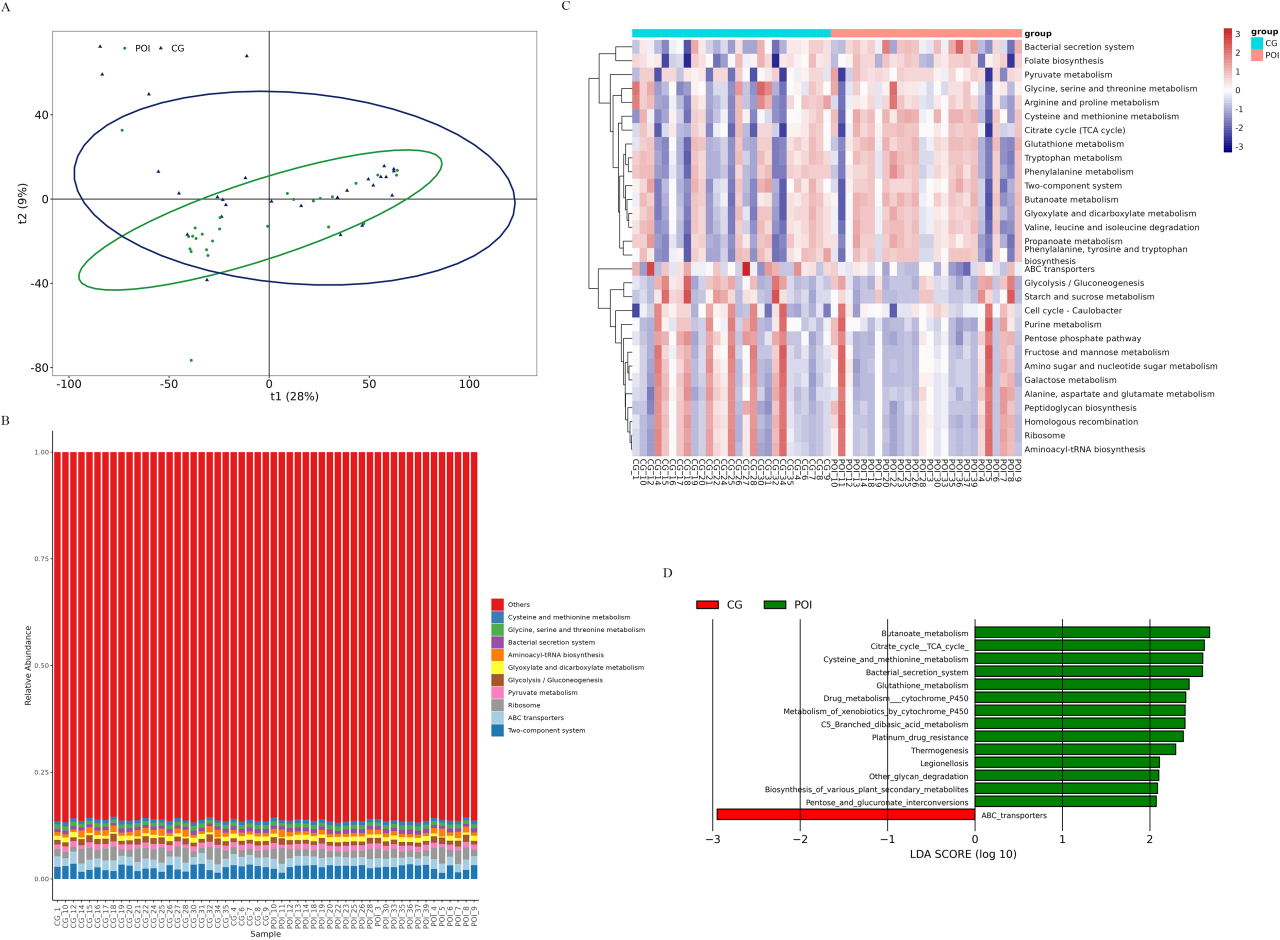
KEGG function prediction of follicular fluid microbial communities in patients with POI and CG. **(A)** Principal Component Analysis (PCA), **(B)** The functional distribution component diagram showed the top 10 KEGG functional pathways. Different colors represented different functional pathways, corresponding to the right legend; the horizontal axis represented samples in each group, and the vertical axis represented the relative richness of each functional pathway. **(C)** The heatmap of functional distribution. The horizontal axis represented samples in each group, and the vertical axis represented different functional pathways, the darker color meaned the higher richness. **(D)** LEfSe analysis based on KEGG function prediction, showing differential functional pathways between the two groups (bar graph). The length of the bar indicated the LDA score, red bars indicated significantly upregulated pathways in the CG group, and green bars indicated significantly upregulated pathways in the POI group.

By utilizing PICRUSt software and KEGG functional enrichment analysis, we predicted the functions of microbial communities based on 16S rDNA sequencing data (**Supplementary Table S2** for KEGG enrichment pathways). First, a PCA from the overall perspective revealed significant differences in the functional profiles of the two groups (**Figure 3A**). The top 10 KEGG functional pathways were as follows: two component system, ABC transporters, ribosome, pyruvate metabolism, glycolysis/gluconeogenesis, glyoxylate and dicarboxylate metabolism, aminoacyl tRNA biosynthesis, bacterial secretion system, glycine serine and threonine metabolism, and cysteine and methionine metabolism (**Figure 3B**). A heatmap (**Figure 3C**) illustrates the top 30 KEGG functional pathways in the POI group compared to those in the CG group, with 16 being upregulated and 14 being downregulated. To further identify differential functional pathways between groups, LEfSe analysis (screening criteria: LDA score > 2, p value < 0.05) was employed based on the KEGG functional prediction results (**Figure 3D**; **Supplementary Table S3**), resulting in a total of 15 significantly different pathways in the POI group. Among these genes, 14 were upregulated (bacterial secretion system, cysteine and methionine metabolism, butanoate metabolism, TCA cycle, glutathione metabolism, platinum drug resistance, drug metabolism-cytochrome P450, metabolism of xenobiotics by cytochrome P450, pentose and glucuronate interconversions, C5 branched dibasic acid metabolism, legionellosis, thermogenesis, biosynthesis of various plant secondary metabolites, and other glycan degradation), while only one was downregulated (ABC transporters).

### 3.3 Untargeted metabolomic analysis

This study employed LC–MS/MS to investigate alterations in the follicular fluid metabolites of the two groups. The structure of the metabolites was identified by matching with the metabolite retention time, molecular mass (within a 25 ppm error margin), secondary fragmentation spectra, and collision energy information in the local database, and the identification results were strictly checked and confirmed manually (19, 20). The identification level was above Level 2.

**Figure 4:**
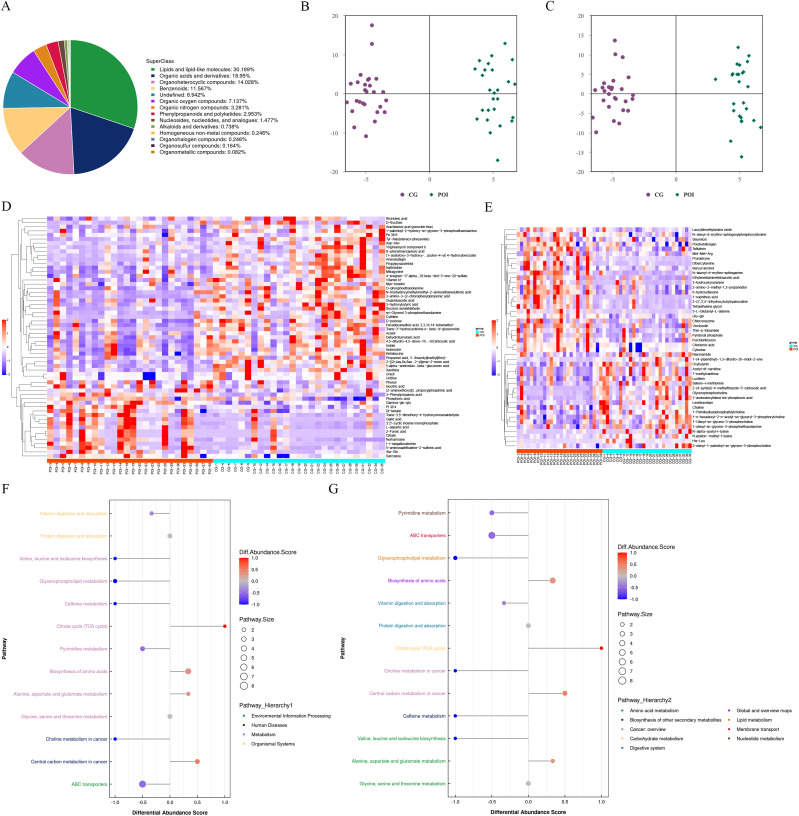
Untargeted metabolomics analysis of follicular fluid in patients with POI and CG. **(A)** Chemical classification and attribution statistics of metabolites. **(B)** OPLS-DA score plot of negative ion mode. **(C)** OPLS-DA score plot of positive ion mode. **(D)** Hierarchical clustering heat map of differential metabolites in negative ion mode. **(E)** Hierarchical clustering heat map of differential metabolites in positive ion mode. **(F)** Differential abundance score (DAS) map of the differential metabolic pathways classified according to Pathway Hierarchy 1. **(G)** DAS map of the differential metabolic pathways classified according to Pathway Hierarchy 2.

After merging the positive and negative ion modes, a total of 1219 metabolites were identified, of which 692 and 527 were identified in the positive and negative ion modes, respectively. According to their chemical taxonomy classification information, they were classified and statistically counted (**Figure 4A**). The difference between the two groups in the positive and negative ion modes was found to be high using OPLS-DA (**Figures 4B, C**). Sevenfold cross-validation (negative ion mode R2Y= 0.972, Q2 = 0.518; positive ion model R2Y= 0.977, Q2 = 0.552) showed that the model was stable and reliable. The permutation test results (negative ion mode R2 = (0.0, 0.9445), Q2 = (0.0, -0.3754); positive ion mode R2 = (0.0, 0.9542), Q2 = (0.0, -0.3662)) also showed that the model had good robustness, with no overfitting.

Furthermore, based on the screening criteria for differentially abundant metabolites, which included an OPLS-DA VIP > 1 and a P value < 0.05, we identified a total of 105 differentially abundant metabolites (**Figures 4D, E**; **Supplementary Table S4**). Among these genes, 59 were detected in the negative ion mode (14 upregulated and 45 downregulated), while 46 were detected in the positive ion mode (19 upregulated and 27 downregulated). After merging the differentially abundant metabolites from both modes, we performed functional annotation using the KEGG database. The differential abundance score (DAS) results (**Figures 4F, G**; **Supplementary Table S5**) revealed that there were 11 differential metabolic pathways in the POI group. Specifically, four pathways were upregulated and were mainly enriched in central carbon metabolism in cancer, alanine, aspartate, and glutamate metabolism, biosynthesis of amino acids, and TCA cycle. On the other hand, seven pathways were downregulated and were mainly involved in ABC transporters, choline metabolism in cancer, pyrimidine metabolism, vitamin digestion and absorption, caffeine metabolism, glycerophospholipid metabolism, and valine, leucine and isoleucine biosynthesis. These findings suggested significant changes in the metabolic profile of follicular fluid in patients with POI.

### 3.4 Conjoint analysis of 16S rDNA amplicon sequencing and metabolites in follicular fluid

**Figure 5:**
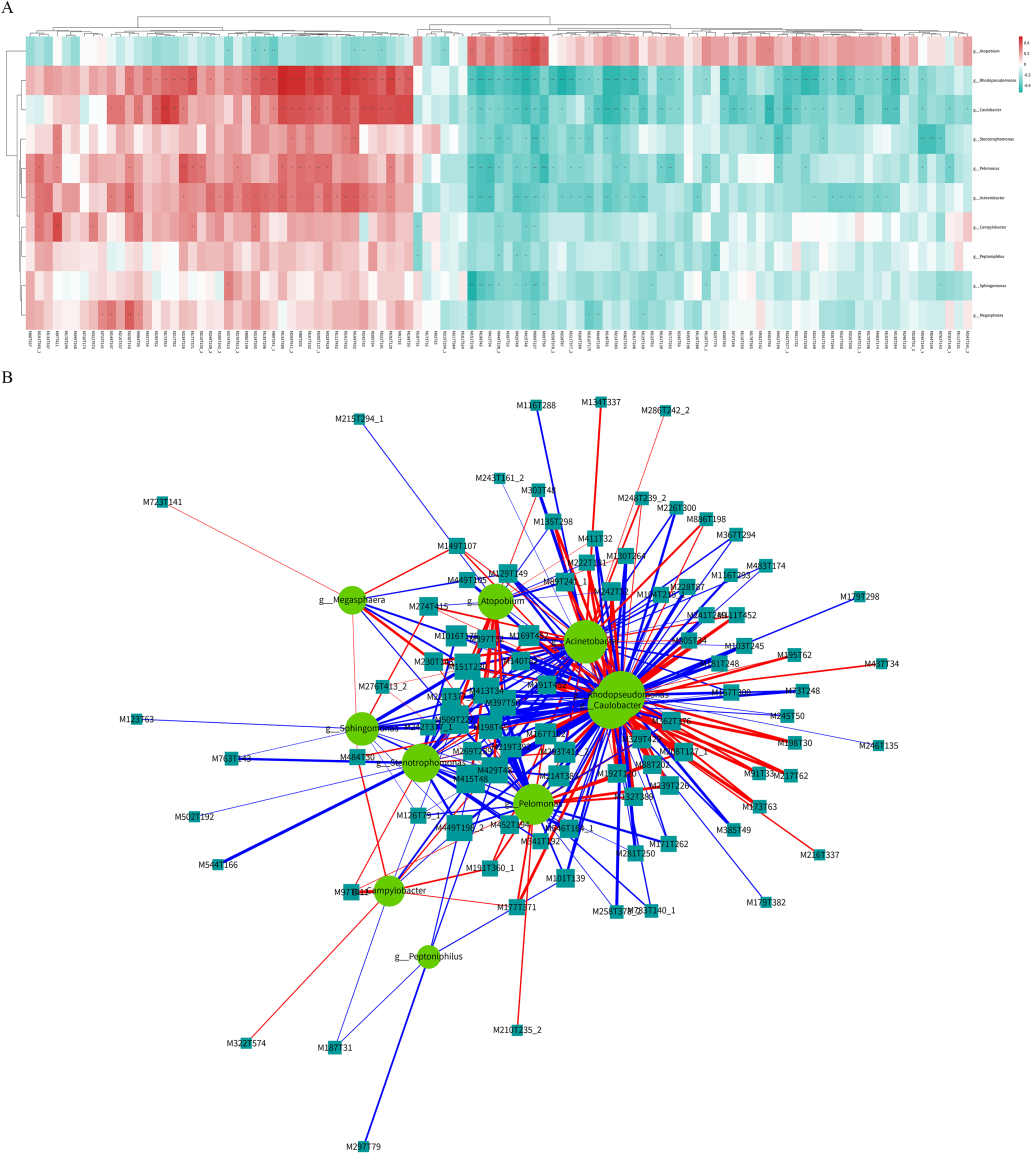
Co-analysis of differential microbial communities and differential metabolites of follicular fluid in patients with POI and CG. **(A)** Hierarchical cluster heatmap of spearman correlation analysis. Each row represented a differential microbial community, and each column represented a differential metabolite. The correlation coefficient (r) was represented by color. r>0 indicated positive correlation, represented by red, r<0 indicated negative correlation, represented by blue, the darker color meaned the stronger correlation. p value<0.05 was indicated by *; p value<0.01 was indicated by **; p value<0.001 was indicated by ***. **(B)** Spearman correlation analysis network diagram. Circles represented differential microbial communities, and rectangles represented differential metabolites. The color of the line represented negative correlation in blue and positive correlation in red, and the thickness of the line was proportional to the absolute value of the r. The size of the node was positively correlated with its degree.

The POI and CG groups exhibited significant differences in microbial diversity and metabolic levels. A total of 10 differential microbial communities and 105 differentially abundant metabolites were identified. Spearman’s correlation analysis was employed to generate a hierarchical cluster heatmap using the R 3.4.2 heatmap package (**Figure 5A**; **Supplementary Tables S6**, **S7**). Additionally, a correlation network map was constructed using Cytoscape 3.5.1 software (**Figure 5B**; **Supplementary Table S8**). A total of 295 pairs of significantly correlated microbial communities and metabolites were identified, including 117 pairs with a significantly positive correlation (p <0.05), 178 pairs with a significantly negative correlation (p <0.05), 96 pairs with a more significant correlation (0.001<p <0.01), and 28 pairs with an extremely significant correlation (p <0.001).

The 28 pairs with extremely significant correlations (**Table 2**) included five microbial communities and 26 differentially abundant metabolites. The five differential microbial communities were mainly g-Sphingomonas, g-Rhodopseudomonas, g-Caulobacter, g-Pelomonas, and g-Stenotrophomonas. Additionally, the corresponding metabolites were classified into the following main categories: imidazolopyrimidines, carboxylic acid and its derivatives, organic sulfonic acid and its derivatives, organic nitrogen compounds, steroids and steroid derivatives, glycerophospholipids, organic oxygen compounds, organic phosphoric acid and its derivatives, azoles, quinoline and its derivatives, benzene and substituted derivatives, prealcoholic lipids, phenols, naphthalenes, and fatty acyl and pyrimidine nucleotides.

**Table 2:** Extremely significant correlation between 28 pairs of differential microbial communities and metabolites.

Genus	Metabolites	Coefficient	P value	Lable
g_Sphingomonas	Xanthine	-0.5302	0.00004449	neg
g_Rhodopseudomonas	L-aspartic acid	0.5229	0.00005897	pos
	N-tris(hydroxymethyl)methyl-2-aminoethanesulfonic acid	-0.5188	0.00006893	neg
	Ethylenediaminetetraacetic acid	0.4980	0.0001478	pos
	2-amino-2-methyl-1,3-propanediol	0.4942	0.0001693	pos
	Arenobufagin	-0.4916	0.0001853	neg
	Luciferin	-0.4791	0.0002841	neg
	sn-Glycerol 3-phosphoethanolamine	-0.4786	0.0002891	neg
	3-hydroxykynurenine	0.4781	0.0002941	pos
	Citrate	0.4739	0.0003379	pos
	O-phosphoethanolamine	-0.4729	0.0003497	neg
	3-amino-3-(2-chlorophenyl)propionic acid	-0.4722	0.0003567	neg
	2-(4-pyridyl)-4-methylthiazole-5-carboxylic acid	-0.4684	0.0004046	neg
	4,5-dihydro-4,5-dioxo-1h-pyrrolo[2,3-f]quinoline-2,7,9-tricarboxylic acid	-0.4644	0.0004609	neg
	Gallic acid	0.4573	0.0005755	pos
	Seleno-l-methionine	-0.4485	0.0007576	neg
	(1-acetyloxy-3-hydroxy-6,8a-dimethyl-7-oxo-3-propan-2-yl-2,3a,4,8-tetrahydro-1h-azulen-4-yl) 4-hydroxybenzoate	-0.4463	0.0008091	neg
	Trans-3,5-dimethoxy-4-hydroxycinnamaldehyde	0.4434	0.0008823	pos
	Isoleucine	-0.4422	0.0009153	neg
	Naltrindole	-0.4397	0.0009873	neg
g_Caulobacter	1-naphthoic acid	0.4736	0.0003410	pos
	O-phosphoethanolamine	-0.4723	0.0003555	neg
	Arachidonic acid (peroxide free)	-0.4717	0.0003630	neg
	sn-Glycerol 3-phosphoethanolamine	-0.4657	0.0004406	neg
	Cytidine	-0.4394	0.0009968	neg
g_Pelomonas	Tyr-Ala(dstereo)-phe(amide)	-0.4499	0.0007251	neg
g_Stenotrophomonas	1-Oleoyl-sn-glycero-3-phosphocholine	-0.4458	0.0008223	neg
	2-[(2r,4as,8s,8as)-8-[2-[(4as,7r,8ar)-7-(1-carboxyethenyl)-1-hydroxy-4a-methyl-2-oxo-6,7,8,8a-tetrahydro-5h-	-0.4401	0.0009743	neg
	naphthalen-1-yl]ethyl]-4a-methyl-7-oxo-1,2,3,4,8,8a-hexahydronaphthalen-2-yl]prop-2-enoic acid			

**Figure 6:**
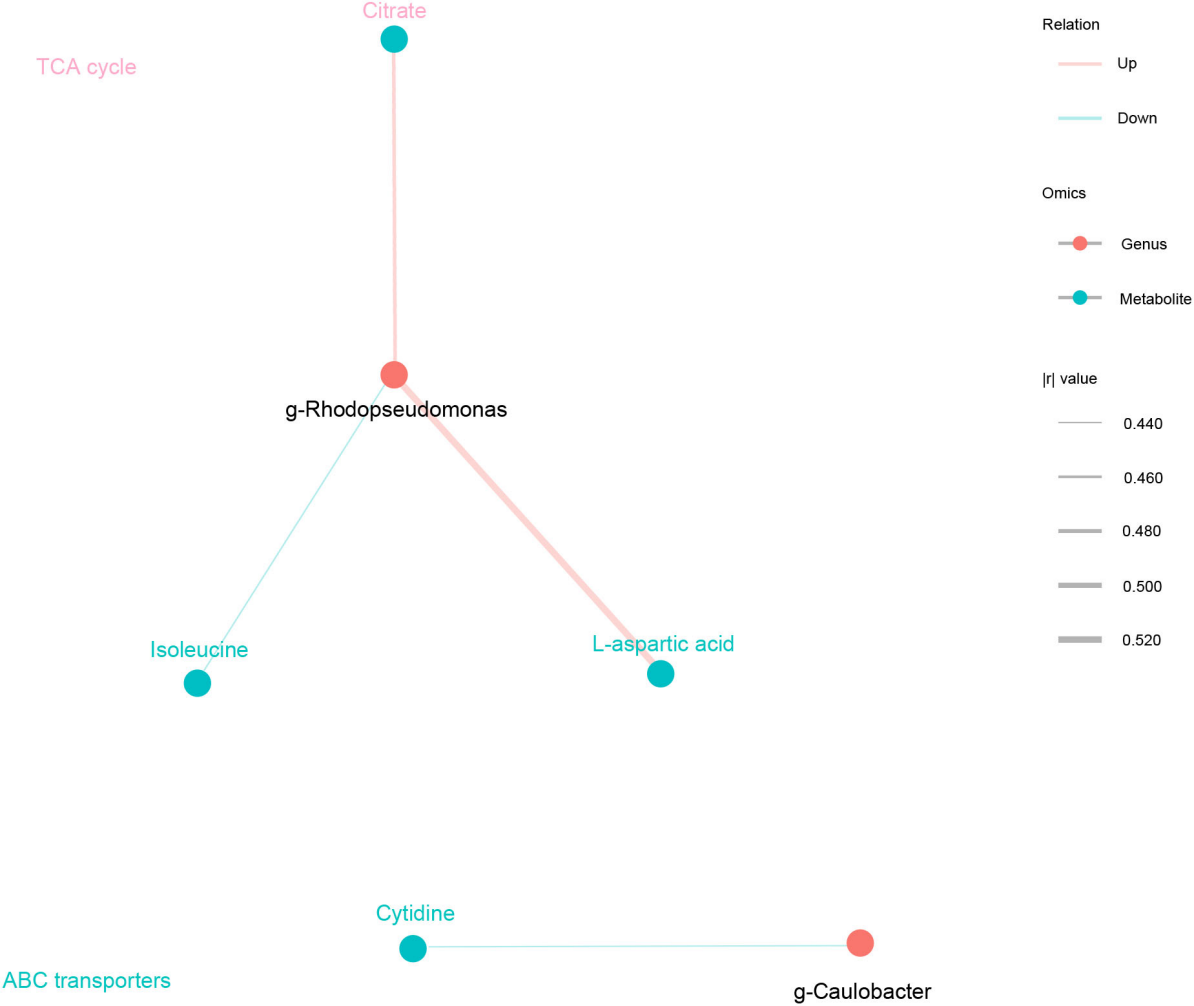
Key microbial communities and metabolites that regulate the occurrence and development of POI. Spearman correlation analysis network diagram. Black circles represented key microbial communities, and light blue circles represented key metabolites. The color of the line represented negative correlation in blue and positive correlation in red, and the thickness of the line was proportional to the absolute value of the r. The size of the node was positively correlated with its degree. The metabolite is the same color as the corresponding pathway.

Finally, based on KEGG functional prediction of microbial communities and the differential metabolic analysis that regulate POI, two major differential microbial communities were identified: g-Rhodopseudomonas and g-Caulobacter. Two common metabolic pathways were also found: ABC transporters and TCA cycle. The ABC transporter pathway mainly involved eight differentially abundant metabolites, namely, cytidine, D-fructose, isoleucine, L-aspartic acid, myo-inositol, phosphoric acid, uridine and choline. TCA cycle pathway mainly involved two differentially abundant metabolites, namely, citrate and isocitric acid. Among them, four major differentially abundant metabolites were identified: three metabolites in the ABC transporter pathway (cytidine, isoleucine, and L-aspartic acid) and one metabolite in TCA cycle pathway (citrate) (**Figure 6**, **Table 3**). In conclusion, downregulation of ABC transporters and upregulation of TCA cycle might be the key metabolic pathways for the occurrence and development of POI, with g-Rhodopseudomonas and g-Caulobacter as key microbial communities and L-aspartic acid, citrate, isoleucine, and cytidine as key metabolites.

**Table 3:** Key microbial communities and metabolites that regulate the occurrence and development of POI.

Genus	Metabolites	Coefficient	P value	LABEL
g_Rhodopseudomonas	L-aspartic acid	0.5229	0.00005897	pos
	Citrate	0.4739	0.0003379	pos
	Isoleucine	-0.4422	0.0009153	neg
g_Caulobacter	Cytidine	-0.4394	0.0009968	neg

## 4 Discussion

The incidence of POI is 3%, with 2-3% of cases occurring in individuals aged 30-40 years and 0.1% occurring in individuals younger than 30 years. In recent years, the incidence of this disease has increased, which not only seriously affects women’s reproductive health but also has certain effects on glucose and lipid metabolism, bone metabolism, the nervous system, and the cardiovascular system (3, 21, 22). The etiology of POI is complex, and its pathogenesis is still unclear. This study was the first to analyze the correlation between microbial communities and the metabolome of POI patients based on 16S rDNA amplicon sequencing and nontargeted metabolic technology. The results showed that the richness and diversity of the microbial communities and metabolome in the follicle fluid of POI patients changed significantly. Downregulation of ABC transporters and upregulation of TCA cycle might be the key metabolic pathways involved in the development of POI.

Some studies have shown that the composition of the intestinal and vaginal microbiota can affect ovarian function. Changes in microbiota imbalance are related to reproductive endocrine hormones such as estrogen, FSH, LH, and AMH, which are also related to autoimmune POI (9, 21, 23). Wu et al. reported that the phylum Bacteroidetes and the genera Butyricimonas, Dorea, Lachnobacterium, and Sutterella in the gut microbiota were significantly enriched in women with POI (9). Jiang et al. reported that the gut microbiota and serum metabolites of POI patients significantly changed, with an increase in the richness of the genus Eggerthella and a positive correlation with serum transforming growth factor (TGF)-beta1 levels (11). The vaginal microbiota of POI patients also changes significantly, including an increase in the abundance of Actinobacteria, Atopobium and Gardnerella and a decrease in the abundance of Bifidobacterium (24). These changes are closely related to ovarian reserve reduction, endocrine disorders, and menopausal syndrome symptoms. As the direct microenvironment for the growth and development of oocytes, it is still unclear whether there is a correlation between the microbial communities in follicular fluid and POI. This study revealed that the structure and richness of the microbial communities in the follicle fluid of POI patients changed significantly. LEfSe analysis indicated that there were 22 differential microbial communities at different levels between the two groups, including 10 differential communities at the genus level. PCA showed that there were differences in the overall functions of the microbial communities between the two groups. LEfSe analysis revealed 15 differential functional pathways in the POI group. Notably, 14 of these pathways were upregulated. Conversely, there was a single downregulated pathway: ABC transporters. It is worth highlighting that ABC transporters, the bacterial secretion system, and cysteine and methionine metabolism were among the top 10 KEGG functional pathways in terms of richness.

Patients with POI are prone to metabolic disorders such as lipid and carbohydrate metabolism, which can lead to long-term complications such as cardiovascular disease and osteoporosis, seriously affecting women’s reproductive health and quality of life. However, the metabolic changes leading to POI are not fully understood, and few studies have described the abnormal metabolomic characteristics of POI patients. Some studies have confirmed that there are multiple differentially abundant metabolites in the plasma of POI patients, involving pathways such as caffeine metabolism and ubiquinone and other terpenoid-quinone biosynthesis and plasma arachidonoyl amide, 3-hydroxy-3-methylbutanoic acid, dihexyl nonanedioate, 18-HETE, cystine, and PG (16:0/18:1), which are correlated with ovarian reserve (25). In the follicular fluid of patients with DOR, 12 upregulated and 32 downregulated differentially abundant metabolites were found, mainly involving amino acids, indoles, nucleosides, organic acids, steroids, phospholipids, fatty acyls, and organic oxygen compounds. These metabolites mainly participate in pathways such as aminoacyl-tRNA biosynthesis, tryptophan metabolism, pantothenate and coenzyme A biosynthesis, and purine metabolism (26). This study used an LC–MS/MS method to identify changes in follicular fluid metabolites of POI. The OPLS-DA results revealed that the discrimination between the positive and negative ion modes in both groups was high, with significant intergroup differences. Based on the screening criteria OPLS-DA VIP > 1 and P value < 0.05, a total of 105 differentially abundant metabolites were found, with 59 in negative ion mode (upregulated 14, downregulated 45) and 46 in positive ion mode (upregulated 19, downregulated 27). The DAS analysis revealed 11 differential metabolic pathways in the POI group, four of which were upregulated and seven of which were downregulated. These findings confirmed that the metabolic pattern of follicular fluid in POI patients changed.

Next, Conjoint analysis was conducted between the 10 differential microbial communities and 105 differentially abundant metabolites. A total of 295 pairs of significantly correlated differential communities and metabolites were identified. Among them, 117 pairs had significant positive correlations (p < 0.05), 178 pairs had significant negative correlations (p < 0.05), and 96 pairs had moderately significant correlations (0.001 < p < 0.01). Additionally, 28 pairs showed extremely significant correlations (p < 0.001), involving five differential microbial communities and 26 differentially abundant metabolites. Finally, based on the prediction of microbial community metabolic functions and untargeted metabolic pathway analysis, two main differential microbial communities were identified, namely, g-Rhodopseudomonas and g-Caulobacter. These communities were found to be involved in two common metabolic pathways, namely, ABC transporters and TCA cycle. Four primary differentially abundant metabolites were identified in these pathways: three metabolites from ABC transporters (cytidine, isoleucine, and L-aspartic acid) and one metabolite from TCA cycle (citrate).

ABC transporters are primarily responsible for the transport of various metabolic substrates, including nutrients, amino acids, nucleotides, ions, and metabolites. This pathway controls the entry and exit of substances from cell membranes to maintain normal physiological cellular functions. It might also play a regulatory role in ovarian reserve function by affecting the development and maturation of oocytes, the synthesis and secretion of ovarian hormones, and the apoptosis of ovarian cells. ABC transporter P-glycoprotein (P-gp, encoded by ABCB1) and breast cancer-related protein (BCRP, encoded by ABCG2) are closely related to ovarian reserve function. P-gp is present in both the lumen and the outer membrane of ovarian endothelial cells and granulosa cells, and BCRP is located on the lumen side of ovarian vascular endothelial cells. P-gp responded strongly to LH and progesterone, indicating that this transporter plays an important role in regulating oocyte formation and steroidogenesis (27). P-gp and BCRP in ovarian endothelial cells can prevent potentially harmful foreign substances (including drugs and environmental toxins) from entering the body, protecting the ovaries from toxins and metabolite accumulation (28). Therefore, we speculated that ABC transporters are highly important for maintaining the microenvironment of the ovary and protecting the ovary from harmful substance accumulation.

In addition, TCA cycle was the final metabolic pathway for three major nutrients. It also serves as a junction between carbohydrate, lipid, and amino acid metabolism. During the quiescent and early stages of the ovarian follicle, oocytes primarily rely on TCA cycle and glycolysis for energy metabolism. TCA cycle is an important way for cells to obtain energy, but it can also generate potentially toxic byproducts such as reactive oxygen species (ROS) (29). Under normal conditions, cells possess an antioxidant system to eliminate these ROS and maintain cell health and function. However, if the production of ROS exceeds the capacity of the antioxidant system or its function is compromised, the level of ROS increases, leading to oxidative stress and cellular damage. Therefore, upregulation of TCA cycle pathway might result in excessive accumulation of ROS and contribute to the occurrence of POI.

In conclusion, downregulation of ABC transporters and upregulation of TCA cycle might be crucial metabolic pathways involved in the development of POI. Among these, g-Rhodopseudomonas and g-Caulobacter were key microbial communities, while L-aspartic acid, citrate, isoleucine, and cytidine were key metabolites. Alterations in microbial communities and metabolic pathways in POI patients might serve as novel therapeutic targets. These findings offer new insights for further exploring the pathogenesis of POI. Next, we will further investigate the mechanism underlying POI through animal models by elucidating changes in key microbial communities metabolic pattern. Additionally, we aim to develop new microbial agents that can improve pregnancy outcomes in patients with POI. This introduces a novel therapeutic strategy for POI.

## Data Availability

The original contributions presented in the study are included in the article/**Supplementary Material**. Further inquiries can be directed to the corresponding author.
